# Development, validation, and cost-effectiveness analysis of an AI-assisted three-tiered glaucoma screening model in a community-based setting: protocol for a cluster randomized controlled trial

**DOI:** 10.3389/fpubh.2026.1845337

**Published:** 2026-05-25

**Authors:** Xiangyu Fu, Jiaying Zhang, Shanming Jiang, Xi Liu, Haotian Xiang, Xianjie Yu, Meng Wang, Yutong Liu, Li Tang

**Affiliations:** 1 Department of Ophthalmology, West China Hospital, Sichuan University, Chengdu, China; 2 Research Laboratory of Ophthalmology and Vision Sciences, Eye Research Institute, West China Hospital, Sichuan University, Chengdu, China; 3 Department of Ophthalmology, The Second People’s Hospital of Yibin, Yibin, China

**Keywords:** glaucoma, three-tiered screening model, AI-assisted, community, intraocular pressure, fundus photography, cluster randomized controlled trial

## Abstract

**Introduction:**

Glaucoma is the leading irreversible blinding eye disease worldwide and the global prevalence of glaucoma for individuals aged 40–80 years is estimated as 3.54%. Early screening and prevention wherever possible are essential interventions of chronic disease management for glaucoma, but there is currently a lack of a recognized glaucoma screening model for Chinese population. Therefore, this study intends to construct and validate an artificial intelligence (AI)-assisted three-tiered glaucoma screening model based on the community population and assess its cost-effectiveness.

**Methods and analysis:**

This is a community population-based cluster randomized controlled trial with a minimum of 6-year follow-up. A three-tiered glaucoma screening strategy appropriate for Chinese individuals will be established, including a high-risk model questionnaire (primary screening), ophthalmic examinations (secondary screening, combined with physician-based and AI-assisted image interpretation), and definitive diagnosis by tertiary hospitals (tertiary screening). The participants of each community will be randomly divided into three groups (simple cluster randomization): no screening group, routine screening group, and tiered screening group. Participants in the no screening group will receive regular glaucoma-related health education, structured annual follow-up for symptom monitoring and recording of external ophthalmology visits, and a comprehensive ophthalmic screening at the end of the study to compensate for the absence of regular screening. A total of 28,275 participants (9,425 per group) will be recruited, allowing for an anticipated loss-to-follow-up rate of 20%. All primary outcome measures will be analyzed on a per-participant basis. The main endpoint is the glaucoma detection rate, with sensitivity and specificity of the screening as additional primary outcomes. Secondary outcomes involve comparative analyses of the cost-effectiveness of different screening strategies and the diagnostic accuracy of physician-based versus AI-assisted image interpretation.

**Ethics and dissemination:**

The protocol has been approved by the Biomedical Ethics Review Committee, West China Hospital of Sichuan University [2025(1021)]. All the participants will be required to afford signed informed consent. The study results will be presented at scientific meetings and published in a peer-reviewed journal.

**Clinical trial registration:**

https://www.chictr.org.cn/showproj.html?proj=281642, identifier (ChiCTR2500107852).

## 1 Introduction

Glaucoma is the leading cause of irreversible blinding worldwide and continues to threaten global visual health. The global prevalence of glaucoma among adults aged 40–80 years is estimated to be 3.54%, affecting approximately 80 million people (1, 2). Of these, 10% of patients with primary open-angle glaucoma (POAG) and 25% of those with primary angle-closure glaucoma (PACG) are bilaterally blind (3, 4). In China, the number of glaucoma patients has surpassed 21 million, the highest of any country, and more than 70% of cases are diagnosed at intermediate or advanced stages, or even after monocular blindness has already occurred (5). With population aging, the burden of glaucoma continues to rise. The deterioration in quality of life caused by visual impairment exceeds that associated with many other forms of disability, and glaucoma-related visual loss diminishes the capacity for independent mobility and daily living (6), which in turn has direct and indirect consequences for socioeconomic development and increases the national healthcare burden. Indeed, glaucoma is a critical public health challenge in an aging society.

Although the visual function damage caused by glaucoma is irreversible, it can still be prevented and controlled through early detection (4). If primary glaucoma, including POAG and PACG, is detected and diagnosed before the onset of subjective symptoms, timely and effective intraocular pressure (IOP)-lowering treatment can avoid blindness and substantially alleviate the burden on patients. Nevertheless, owing to the insidious nature of early-stage glaucoma, most patients are already at moderate or advanced stages when first diagnosed. Previous data from China showed that at initial diagnosis, 25% of patients had visual acuity worse than 0.3 (Snellen equivalent of 20/63), and 15% had already progressed to monocular blindness (7, 8). Therefore, early screening, early prevention, early diagnosis, and early treatment are essential components of chronic disease management for glaucoma.

However, a universally recognized glaucoma screening model is still lacking. Low screening uptake, high costs, and heavy reliance on specialist physicians remain the principal barriers to effective screening (9). Consequently, how to define the target population for screening (more accurate screening population) and which screening protocol to adopt (more feasible) are important questions that warrant further investigation. In developed countries, where POAG is the predominant form (3, 10) and is characterized by a relatively low incidence, gradual progression, and a diagnostic process often requiring comprehensive examinations, high primary eye care coverage, well-established referral pathways from optometrists to ophthalmologists, and generally lower population density facilitate more efficient healthcare delivery with less resource dispersion. Under these conditions, screening targeting high-risk groups is preferred over population-based screening, as the latter has been shown to be less cost-effective (11). For instance, annual glaucoma screening for high-risk populations has been incorporated into health insurance in the United States, contributing to a reduction in glaucoma-related blindness (12). In contrast, in developing countries and economically disadvantaged regions, limited medical resources are largely allocated to treating patients with intermediate and advanced glaucoma. In China, given the high population density and insufficient primary eye care coverage, implementing population-wide screening for chronic diseases at the community level is therefore particularly crucial.

At present, despite the fact that the Chinese Glaucoma Guideline (2020) has recommended slit lamp microscopy, IOP measurement, and fundus examination as routine glaucoma screening methods (5), the cost-effectiveness of these approaches in screening remains uncertain. Consequently, there is also an urgent need to develop a glaucoma screening model that is cost-effective, scalable, and suited to the national context. Furthermore, rapid economic and technological advances have facilitated the widespread adoption of telemedicine and artificial intelligence (AI), offering innovative approaches to glaucoma screening (13). Specifically, AI-assisted diagnosis and follow-up can markedly improve screening efficiency and quality, reduce dependence on specialists, and save considerable time and financial resources, potentially leading to a more effective, convenient, and cost-effective screening model (14–17).

Therefore, this study intends to conduct a cluster randomized controlled trial with a 6-year follow-up to establish and validate an AI-assisted three-tiered glaucoma screening model based on the community population, assess its cost-effectiveness, and evaluate the performance of AI-assisted diagnostic tools, thereby generating evidence to inform future optimization.

## 2 Methods and analysis

### 2.1 Objectives

Main objectives: (1) To construct and validate a three-tiered glaucoma screening model based on the community population; (2) Preliminary evaluation of the cost-effectiveness of the screening model to provide basic data for subsequent in-depth health economics analysis.

Secondary objectives: (1) To develop an evaluation tool of screening for populations at high risk of glaucoma; (2) To evaluate the application effect of AI-assisted diagnostic tools to provide a basis for subsequent optimization.

### 2.2 Study design

This is a community population-based cluster randomized controlled trial with a 6-year follow-up conducted in several community healthcare centers in Yibin City, Sichuan Province, China. This study will commence with the construction and validation of a preliminary glaucoma screening tool to identify high-risk populations in China. The initial phase involves the formation of a comprehensive item pool through a systematic literature review focusing on glaucoma risk factors, early symptoms, and existing screening instruments, supplemented by structured discussions with glaucoma specialists to capture clinically relevant indicators not fully reflected in the published literature. Integrating evidence from both sources, an initial item pool will be constructed, covering domains such as clinical presentations, medical and family history, medication use, lifestyle habits, and glaucoma awareness.

Subsequently, a Delphi expert consultation process will be conducted with 10–15 national glaucoma specialists representing clinical, epidemiological, and public health expertise. Over 2–3 rounds of anonymous scoring and feedback, panelists will rate each candidate item on importance, relevance, and feasibility using a five-point Likert scale. Item retention will be guided by predefined threshold criteria based on mean importance scores, full-score frequencies, and coefficients of variation. Specifically, items with a mean score ≥ the average, a full-score frequency ≥ the average, and a coefficient of variation ≤ the average will be retained. Additional refinement through expert consensus will be applied to merge redundant items and clarify ambiguous wording. After adaptation for linguistic accessibility and clinical applicability, the final set of items will be determined upon completion of the Delphi process.

Finally, the refined tool will undergo final item selection and rigorous reliability and validity testing using cross-sectional data from the Ophthalmology Department of West China Hospital, Sichuan University. As a result, the finalized screening tool (a well-designed questionnaire) will be determined and employed for screening within the target population in this study.

This screening tool will underpin a proposed three-tiered strategy for glaucoma screening. Tier 1 Screening (Primary Screening): The pre-designed risk assessment questionnaire is administered within the community to identify high-risk individuals. Tier 2 Screening (Secondary Screening): These high-risk individuals undergo a combination of screening tests, including visual acuity, IOP measurement, fundus photography, and ocular optical biometry, which will be conducted at community healthcare or primary care centers. This stage is augmented by AI-assisted image interpretation to identify subjects suspected of having glaucoma. Tier 3 Screening (Tertiary Screening): A definitive diagnosis of glaucoma is established within the ophthalmology department of a tertiary hospital.

In the Tier 2 screening stage, fundus photographs will be interpreted using the Airdoc glaucoma-specific AI automatic screening system, which is built on a deep convolutional neural network algorithm employing an integrated multi-task learning architecture for simultaneous segmentation and classification. Standardized and traceable glaucoma grading decision rules have been established and fully integrated into the screening workflow. The system automatically performs image preprocessing, precise optic disc detection, quantitative analysis of optic disc morphology, segmentation of the optic cup and retinal nerve fiber layer defect (RNFLD) regions, and identification of glaucomatous lesions including localized RNFLD, diffuse RNFLD, optic disc hemorrhage, and optic cup notching. Moreover, it also calculates horizontal and vertical cup-to-disc ratios, ultimately generating a quantitative glaucoma risk score and a graded recommendation.

This AI system has been trained and internally validated on a large-scale fundus image dataset from Chinese PLA General Hospital, achieving a mean area under the receiver operating characteristic curve (AUC) of 0.941 for optic disc segmentation, 0.874 for optic cup segmentation, and 0.893, 0.901, 0.969, and 0.892 for the classification of the four aforementioned lesions, respectively (18). It has undergone independent external dataset validation and multicenter prospective clinical performance evaluation, with both algorithmic stability and generalizability systematically assessed. Within our screening workflow, all fundus photographs will first be independently interpreted by ophthalmologists at the level of attending physician or higher, while simultaneously undergoing automated image interpretation and risk stratification by the AI system. Then, the human and AI interpretations will be compared. In cases of disagreement, the images will be referred to a glaucoma specialist for final adjudication, and the specialist’s judgment will serve as the reference standard. This forms a standardized screening pattern combining AI-assisted initial screening with physician review for final confirmation.

All participants who enter the Tier 3 screening stage will undergo further examinations for definitive diagnosis, including standard automated perimetry for visual field testing, optical coherence tomography (OCT) imaging of retinal nerve fiber layer (RNFL) thickness, and gonioscopy to distinguish open-angle from angle-closure glaucoma. The final diagnosis will be made independently by two glaucoma specialists following a comprehensive review of all clinical data, with reference to internationally accepted diagnostic criteria for glaucoma, namely the presence of characteristic glaucomatous optic neuropathy with corresponding visual field defects, after excluding other ocular or neurological conditions that may cause optic neuropathy. Any diagnostic disagreement between the two specialists will be resolved by a third senior glaucoma expert who will make a final determination. To correct for verification bias, a random sample of 10% of participants who are screen-negative will also be recalled to undergo the same reference standard examination. Sensitivity and specificity of the two screening strategies will be calculated based on all screen-positive participants and the randomly selected screen-negative subset, with appropriate weighting to account for the sampling fraction.

The study is scheduled to begin patient enrollment in October 2025, with follow-up and final data collection expected to be completed in December 2032.

### 2.3 Inclusion, exclusion, and termination criteria

Inclusion criteria: Community population aged 18 years or older with informed consent. The threshold of 18 years is selected to encompass a broader spectrum of glaucoma types beyond typical age-related cases, such as juvenile and secondary glaucoma.

Exclusion criteria: (1) Inability to complete the questionnaire or comply with long-term follow-up; (2) Inability to undergo IOP measurement or fundus photography due to poor cooperation, low compliance, or coexisting ocular pathologies (such as corneal opacities, advanced cataracts, or vitreous opacities); (3) Presence of other ocular conditions that could confound the definitive diagnosis of glaucoma, such as retinitis pigmentosa or status post panretinal photocoagulation (PRP).

Termination criteria: When study participants in any group are diagnosed with glaucoma through screening or self-visit, they will be recorded and removed from the screening program. These patients will be enrolled in a chronic disease follow-up management to monitor disease progression and assess its impact on quality of life.

### 2.4 Grouping schemes

According to the cluster randomized study design, each community serves as a cluster. The statistician uses a computer-generated random sequence to independently and with equal probability assign each cluster to one of the three groups (simple cluster randomization): (1) blank control group (no screening group), (2) routine screening group, and (3) tiered screening group. To set up blank controls can analyze the clinical significance and health economic value of routine screening mode for the early diagnosis and treatment of glaucoma. On this basis, it is sufficient to compare the difference in efficiency and cost-effectiveness between the routine screening mode and the tiered screening mode. Baseline data and follow-up outcomes are collected uniformly for all participants.

**Figure 1:**
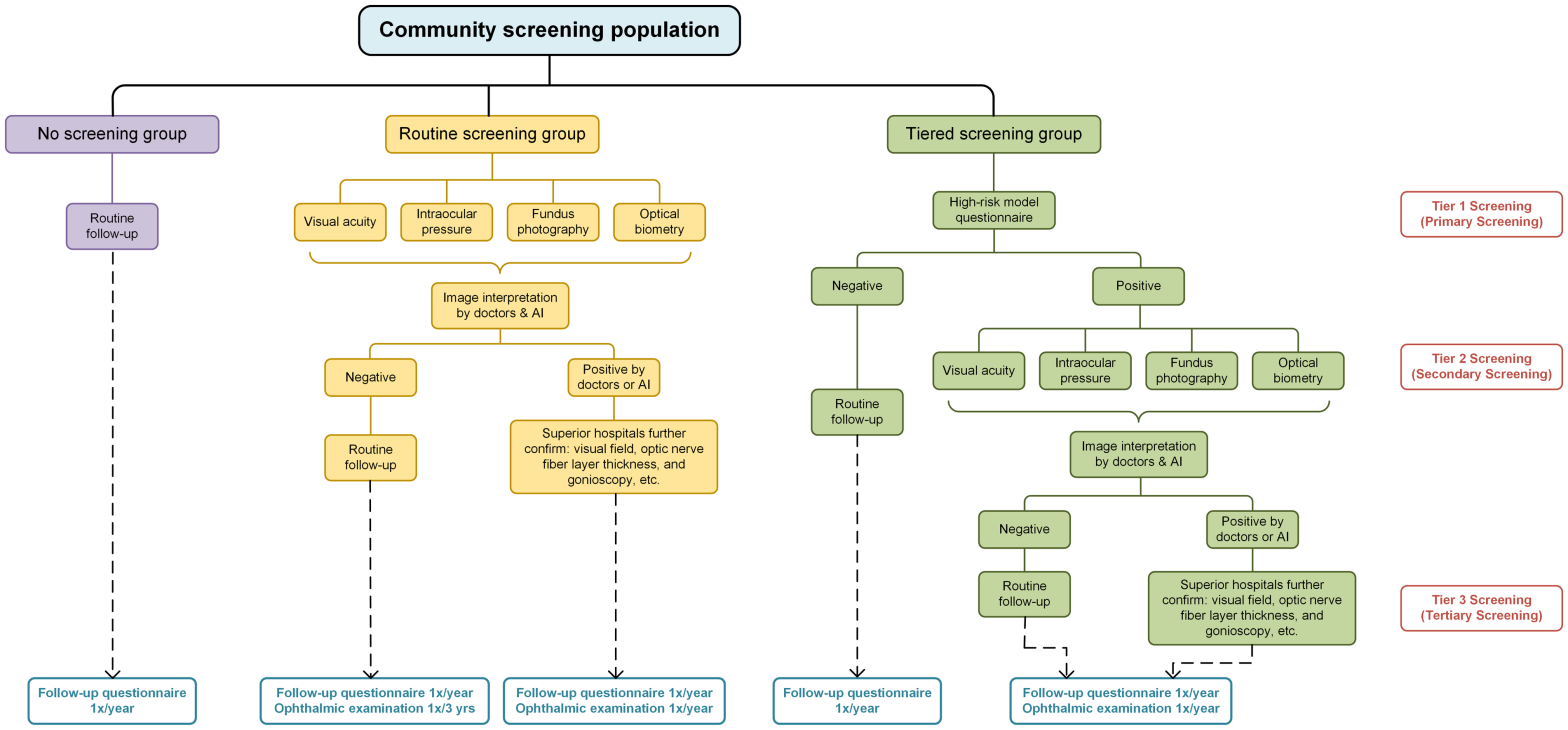
Methodology flowchart of the study. AI, artificial intelligence.

The control group (no screening group) does not undergo any glaucoma screening, and the study subjects can detect and confirm glaucoma through active medical visit or opportunistic screening. The routine screening group is screened with IOP measurement combined with fundus photography to detect glaucoma. The three-tiered screening model designed in this study is adopted in the tiered screening group. It is expected that high-risk populations of glaucoma are identified in the tier 1 screening, who will enter the tier 2 and/or 3 screenings to diagnose or exclude glaucoma (Figure 1).

The screened high-risk populations, suspected patients, and diagnosed glaucoma patients from all the three groups will receive standard glaucoma treatment and AI-assisted intelligent follow-up of chronic disease management, including health education, subsequent visit reminders and medication guidance, so as to expand the coverage and reduce the labor cost of follow-up.

A no-screening control arm is included in this study, in which participants will not receive any ophthalmic examinations actively provided by the study protocol. As the study population consists of community-dwelling individuals rather than confirmed patients, the absence of active screening does not pose additional disease-related risks to the participants. Subjects in the control arm remain free to seek medical attention if symptoms arise or to undergo opportunistic screening through routine healthcare encounters. Therefore, enrollment does not restrict their access to external medical services or affect their standard of care.

To strengthen the ethical justification for the no-screening control group, the following safeguards will be implemented: (1) Regular glaucoma-related health education and disease awareness sessions will be provided to the control group throughout the study period to help participants recognize early symptoms and understand the potential harms of glaucoma, thereby enhancing their capacity for self-monitoring and healthcare-seeking behavior. (2) A structured annual follow-up protocol will be established to collect health status data from the control arm via standardized questionnaires or digital platforms, with particular attention to monitoring symptoms such as visual acuity changes or ocular discomfort, and systematically recording any ophthalmology visits initiated by participants, including the reasons for consultation, examinations performed, and resulting diagnoses. (3) At the end of the study period, a comprehensive ophthalmic screening will be uniformly offered to all participants in the control arm to compensate for the opportunity loss resulting from the absence of regular screening during the study. (4) Periodic reports on study progress and participant health status will be submitted to the Ethics Committee to ensure ongoing adherence to ethical standards, with measures adjusted as necessary based on emerging circumstances.

To address potential parallel non-study eye care, a standardized procedure will be established to systematically collect information on all external ophthalmology encounters across all study arms at each follow-up time point. This will include optometry visits, outpatient consultations, and hospital admissions occurring outside the protocol, along with details of the healthcare facility, conducted examinations, principal findings, and clinical recommendations. These data will be incorporated as important covariates in the statistical analyses to evaluate the potential diluting or confounding effects of parallel care on the estimated effectiveness of the organized screening strategies, and sensitivity analyses will be conducted to assess the magnitude of such effects.

### 2.5 Sample size estimation

The sample size calculation is based on the between-group comparison of glaucoma detection rate, which is the main endpoint of this study. Assuming that the glaucoma detection rate in the routine screening arm would approximate the reported glaucoma prevalence [approximately 3.54% (1, 19)], and that the tiered screening arm, through an optimized screening pathway, would achieve an increased detection rate of 4.54%, corresponding to a difference in detection rate (*Δp*) of 1%. Using this comparison (tiered screening versus routine screening), with a two-sided significance level of α = 0.05 and statistical power of 1 − β = 0.80, the minimum required sample size is calculated to be 6,283 participants per arm. This computation was performed using G*Power (version 3.1.9.7), employing the module for two independent proportions based on Fisher’s exact test.

The sample size is further adjusted for the cluster randomized design by applying a design effect (DEFF). Unlike chronic conditions substantially influenced by behavioral or lifestyle factors, the prevalence of glaucoma is predominantly driven by non-modifiable risk factors such as age and genetic susceptibility. Consequently, between-cluster variation in glaucoma prevalence is expected to be minimal across communities with similar demographic structures. We therefore assumed an intracluster correlation coefficient (ICC) of 0.005 (20) and an average cluster size of 40 participants, which yielded a DEFF of 1.195, approximated as 1.20, resulting in an adjusted requirement of 7,540 participants per arm. In addition, factoring in an anticipated loss-to-follow-up rate of 20%, the final required sample size increases to 9,425 participants per group (totally 28,275 participants in three groups, with *k* = 1).

### 2.6 Follow-ups and outcome variables

Commencing at study initiation, participants will be prospectively followed up annually for a minimum of 6 years. Data will be collected yearly through IOP measurements, fundus photography, and follow-up questionnaires (Table 1). For patients with suspected or confirmed glaucoma, additional data will be collected including ocular optical biometry, RNFL thickness, and automated perimetry. Long-term follow-ups will track annual incidence and blindness rates of glaucoma, and quality of life among glaucoma patients.

**Table 1:** Follow-up schedule.

Groups		Visit number	0	1	2	3	4	5	6
		Visit time (year)	Baseline	1	2	3	4	5	6
No screening group		Questionnaire	×	×	×	×	×	×	×
Routine screening group		Questionnaire	×	×	×	×	×	×	×
		Intraocular pressure	×			×			×
		Fundus photography	×			×			×
Tiered screening group	High-risk population	Questionnaire	×	×	×	×	×	×	×
		Intraocular pressure	×	×	×	×	×	×	×
		Fundus photography	×	×	×	×	×	×	×
		Glaucoma specialist examination	Performed according to the screening results						
	Non-high-risk population	Questionnaire	×	×	×	×	×	×	×
		Intraocular pressure							
		Fundus photography							

All primary outcome measures will be analyzed on a per-participant (per-person) basis. The main endpoint of this study is glaucoma detection rate of the screening protocol, with sensitivity and specificity of the screening included as additional primary outcome measures. Glaucoma detection rate is defined as the proportion of participants in each arm with a final confirmed diagnosis of glaucoma in at least one eye, as determined by the reference standard examination in the screening arms or by passive case-finding through medical records in the control arm. Sensitivity is defined as the proportion of participants with confirmed glaucoma in at least one eye (as determined by the reference standard comprehensive ophthalmic examination) who are correctly identified by the screening protocol. Specificity is defined as the proportion of participants with no glaucoma in either eye who are correctly classified as negative by the screening protocol.

Secondary outcome measures include comparative analyses of the cost-effectiveness of different screening strategies and the diagnostic accuracy of physician-based versus AI-assisted image interpretation in fundus photography for screening glaucoma. The comparison of diagnostic accuracy will be evaluated at the per-participant level and will include sensitivity and specificity, as well as AUC for each interpretation method.

The cost-effectiveness analysis will be conducted from a societal perspective to comprehensively evaluate the resources consumed and health outcomes generated by different screening strategies. The primary economic outcome is the incremental cost-effectiveness ratio (ICER), expressed as the incremental cost per additional glaucoma case detected (based on observed data within the study period), and the incremental cost per quality-adjusted life year (QALY) gained (based on long-term model extrapolation). Cost components will encompass screening-related expenses, including personnel, equipment, consumables, site costs, and AI-assisted interpretation fees; diagnostic confirmation costs following referral, such as OCT and automated perimetry; subsequent treatment and follow-up costs; and direct non-medical and indirect costs, including transportation expenses and productivity losses incurred by patients and their families. Health utility values will be measured prospectively among diagnosed patients during follow-up using the EQ-5D-5L instrument and converted to utility weights using the Chinese value set. Costs and effects within the study period will be obtained through actual data collection, whereas long-term outcomes will be evaluated using a Markov state-transition model simulating the natural history of glaucoma and the long-term trajectory following screening intervention. The model will employ a cycle length of 1 year and a lifetime horizon, and future costs and health outcomes will be discounted to present value at an annual rate of 3%. Uncertainty in the incremental analysis will be assessed through one-way sensitivity analyses and probabilistic sensitivity analysis, and cost-effectiveness acceptability curves will be generated to illustrate the probability that the tiered screening strategy is cost-effective across a range of willingness-to-pay thresholds.

The questionnaire content is divided into a follow-up survey and a high-risk model assessment questionnaire. A WeChat (the most commonly used social software in China) mini-program is designed and developed to facilitate patient-completed data collection, streamlining subsequent follow-up procedures and chronic disease management. The follow-up questionnaire encompasses initial collection of patients’ demographic and clinical characteristics, along with subsequent longitudinal data acquisition, including history of glaucoma confirmed diagnosis, positive symptoms, and results of glaucoma-specific quality of life scale.

The configuration of the high-risk model questionnaire incorporates both retrospective data collection and screening for high-risk populations. Informed by currently established risk factors potentially associated with glaucoma, the questionnaire includes the following domains: age; sex; race/ethnicity; general physical constitution (degree of frailty); personal and family history of glaucoma; type and degree of refractive error; hypertension; diabetes mellitus; vasospastic disorders; migraine; anxiety; depression; Parkinson’s disease; Alzheimer’s disease; pigment dispersion syndrome; pseudoexfoliation syndrome; obstructive sleep apnea syndrome; smoking history; medication history (including steroid use, psychotropic agents, and intraocular anti-VEGF or steroid treatments) and relevant symptoms (21–26).

### 2.7 Quality control, data management and supervision

The quality control and supervision of this study involve the following points:

(1) Prior to study initiation, standardized operating procedures (SOPs) are formulated for visual acuity testing, IOP measurement, fundus photography, and ocular optical biometry at community-based physical examination centers. All examiners will undergo standardized training on these protocols and are required to demonstrate competencies through formal assessment before being permitted to participate in the study.
(2) The original data of examinations including visual acuity, IOP, and optical biometry will be retained for storage in the disease-specific database of the cohort study office, West China Hospital of Sichuan University. At least two researchers complete the form entry and checking work.
(3) Fundus photographs are initially interpreted by experienced physicians holding a rank of attending physician or higher. Then, these assessments are compared with AI-based image analyses. Regarding discrepancy between the human and AI interpretations, the images will be referred to a glaucoma specialist for arbitration, with the expert’s judgment serving as the gold standard.
(4) Automated perimetry and RNFL thickness measurements are served as definitive diagnostic tests, with all results being evaluated and confirmed by a glaucoma specialist whose judgment constitutes the gold standard.
(5) All screening instruments and follow-up questionnaires would be reviewed by dedicated personnel. If there are incomplete filling out of forms or questionable responses, immediate follow-up contacts will be initiated for verification.
(6) A central database is planned to be established and all data can be uploaded to the central database through the researchers in a timely manner, or directly through the inspection equipment via Internet.
(7) The study involves a prolonged follow-up period, during which participant attrition is anticipated. The initial sample size calculation has taken an estimated loss-to-follow-up rate (20%) into consideration by expanding sample size. Furthermore, multiple strategies will be employed to enhance participant compliance, including simplifying questionnaire content to reduce response burden, shortening examination duration, and providing subsidies (financial support provided to cover or offset costs associated with the screening process, such as transportation expenses) as necessary. Additionally, active reattempts are made to contact participants lost to follow-up to determine reasons for withdrawal and minimize attrition wherever possible.

### 2.8 Patient and public involvement no patient or public involvement

### 2.9 Statistical analyses

All statistical analyses will be performed using SPSS V25.0 (IBM, United States) and R software. Continuous variables will be expressed as Mean ± Standard Deviation (SD) or Median with Interquartile Ranges (IQRs), depending on the data distribution, while categorical variables will be presented as frequencies and percentages. Descriptive statistics will be used to summarize the baseline characteristics of each cohort.

Given the cluster randomized design of this study, all between-group comparisons will account for clustering effects. The comparison of the glaucoma detection rate as the primary endpoint will be analyzed using generalized estimating equations (GEE). The GEE model will employ a binomial distribution family and a logit link function, with study group as a fixed effect and community as the clustering variable, adopting an exchangeable working correlation matrix to adjust for within-community correlation. Comparisons of sensitivity and specificity will similarly be conducted using GEE to correct standard errors and confidence intervals (CIs). Cox proportional hazards regression and logistic regression analyses will incorporate cluster as a clustering variable or random intercept to identify risk factors associated with glaucoma incidence. Pearson’s χ^2^ test is utilized only for preliminary comparisons without adjustment for clustering, and final conclusions will be based on results accounting for clustering effects. A two-sided *p* < 0.05 will be considered statistically significant.

For missing data, the pattern, frequency, and possible causes of missingness will first be described. Baseline characteristics of participants with and without complete data will be compared to assess the missing data mechanism (whether missing at random). Primary outcome analyses will be performed using multiply imputed datasets, with complete-case analysis serving as a sensitivity analysis. For participants lost to follow-up after screening, sensitivity analyses using inverse probability weighting will be conducted to evaluate the potential impact of attrition on the estimation of the primary outcomes.

Additionally, the cost-effectiveness analysis will adopt ICER as the economic evaluation measure, with estimated CIs using nonparametric bootstrapping. The sensitivity, specificity, and AUC of physician-based versus AI-assisted image interpretation will be calculated and compared, and differences in AUC will be assessed by the DeLong test or bootstrapping.

## 3 Ethics and dissemination

### 3.1 Ethics approval

This study has been approved by the Biomedical Ethics Review Committee, West China Hospital of Sichuan University [2025(1021)].

### 3.2 Informed consent

Before the enrollment in this study, the investigator is responsible for providing a comprehensive explanation of the study’s purpose, procedures, and potential risks to each participant and/or their legally representatives. Signed informed consent must be obtained following this disclosure. Participants should be explicitly informed that their participation is entirely voluntary, and that they may refuse to participate or withdraw from the study at any stage without facing discrimination or reprisal.

### 3.3 Confidentiality

Clinical data of study subjects will be obtained through routine examination and follow-up, which will be used for statistical analyses and research purposes only. All data will be kept strictly confidential and comply with relevant ethical and privacy protection requirements.

### 3.4 Dissemination policy

The research results are planned to be disseminated at scientific meetings and published in a peer-reviewed journal.

## 4 Discussion

This study aims to conduct a community-based cluster randomized controlled trial to evaluate the feasibility, effectiveness, and cost-effectiveness of a systematic three-tiered screening strategy for glaucoma. Irreversible visual impairment is the hallmark of glaucoma as the leading cause of irreversible blindness worldwide, a tragic outcome largely attributable to delayed diagnosis. Although the necessity of glaucoma screening is widely acknowledged within the academic community, key questions regarding “how to screen,” “where to screen,” and “which populations to target” remain unresolved without a unified optimal pathway. Current hospital-based opportunistic screening models have limited coverage, while some previous population-based studies, despite providing valuable epidemiological data, have encountered major challenges in the feasibility and sustainability of their complex examination protocols (such as requiring all participants to undergo visual field testing) in real-world community settings (27, 28).

The superiority of the three-tiered screening protocol designed in this study lies in its establishment of a more cost-effective and scalable tiered screening model. Previous research tended to employ relatively homogeneous examinations for broad populations (29), which entailed substantial resource consumption and demonstrated insufficient targeting of asymptomatic high-risk individuals. In contrast, this study proposes the use of a structured questionnaire in the tier 1 screening to identify high-risk individuals (e.g., aged > 40 years, with a family history, or with high myopia), thereby significantly narrowing the population base requiring secondary screening, and avoiding the inefficient pattern of conducting expensive examinations on all community residents. This approach aligns with the concept of exploring risk factors in the Singapore Epidemiology of Eye Diseases (SEED) study (30), while our protocol aims to further develop this concept into an operational screening tool, substantially enhancing the positive detection rate of subsequent examinations.

Furthermore, the integration of AI constitutes a pivotal component in enhancing the screening efficiency of this study, significantly bolstering the capacity for rapid and standardized preliminary screening at community healthcare centers. Its practical advantage is demonstrated through the capacity to generate objective and reproducible interpretive results within shortened timeframes, effectively addressing the shortage of ophthalmological specialists in primary care settings and reducing dependence on expert evaluations for large-scale screening initiatives. More importantly, its diagnostic efficacy has been substantiated by several high-quality studies. For instance, one investigation demonstrated that a full AI-based glaucoma screening network exhibits high sensitivity and specificity in real-world testing utilizing fundus images (15). Similarly, a prospective study evaluated an offline, smartphone-based AI system for the detection of referable glaucoma, reporting robust performance comparable to that of glaucoma specialists (31). Consequently, the incorporation of AI into the secondary screening of this protocol can establish a robust technical foundation for achieving widespread early detection of glaucoma in resource-limited settings.

However, several potential limitations warrant consideration, such as selection bias and low follow-up compliance. On the one hand, extensive community-based screening relying on voluntary participation may lead to over-representation of individuals with greater health awareness, potentially resulting in an underestimation of the true prevalence. On the other hand, long-term tracking of visual outcomes may be compromised by loss to follow-up, which could undermine the validity of the effectiveness evaluation.

Overall, this cluster randomized controlled trial is expected to reveal that the three-tiered glaucoma screening model may achieve a higher glaucoma detection rate and greater cost-effectiveness than the routine screening or no screening. Through tiered glaucoma screening, early screening, early prevention, early diagnosis, and early treatment can be efficiently realized, thereby reducing the personal and socio-economic burden and improving the quality of life of patients. AI is anticipated to offer superior efficiency and cost-effectiveness in disease screening and chronic disease follow-up, and further refinement of AI tools may accelerate the translation of scientific advances into practice in the near future. Accordingly, this study plans to establish a feasible AI-assisted tiered glaucoma screening strategy that can be extended and validated in broader regions, providing a foundation for the formulation of glaucoma-related guidelines.
